# Microstructural Characterization of TiC–White Cast-Iron Composites Fabricated by In Situ Technique

**DOI:** 10.3390/ma13010209

**Published:** 2020-01-03

**Authors:** Aida B. Moreira, Ricardo O. Sousa, Pedro Lacerda, Laura M. M. Ribeiro, Ana M. P. Pinto, Manuel F. Vieira

**Affiliations:** 1 Department of Metallurgical and Materials Engineering, University of Porto, R. Dr. Roberto Frias, 4200-465 Porto, Portugal; up201108098@fe.up.pt (A.B.M.); rmsousa@fe.up.pt (R.O.S.); lribeiro@fe.up.pt (L.M.M.R.); 2 CEMMPRE - Centre for Mechanical Engineering, Materials and Processes, University of Porto, R. Dr. Roberto Frias, 4200-465 Porto, Portugal; 3 INEGI - Institute of Science and Innovation in Mechanical and Industrial Engineering, R. Dr. Roberto Frias, 4200-465 Porto, Portugal; 4 FERESPE - Fundição de Ferro e Aço Lda., Vila Nova de Famalicão, 4760-485 Fradelos, Portugal; pedrolacerda@ferespe.pt; 5 CMEMS - Center for MicroElectroMechanics Systems, Department of Mechanical Engineering, University of Minho, Campus de Azurém, 4800-058 Guimarães, Portugal; anapinto@dem.uminho.pt

**Keywords:** casting process, high-chromium white cast iron, in situ synthesis, local reinforcement, metal matrix composite, microstructural characterization, titanium carbide

## Abstract

High-chromium white cast-iron specimens locally reinforced with TiC–metal matrix composites were successfully produced via an in situ technique based on combustion synthesis. Powder mixtures of Ti, Al, and graphite were prepared and compressed to fabricate green powder compacts that were inserted into the mold cavity before the casting. The heat of the molten iron causes the ignition of the combustion reaction of the reactant powders, resulting in the formation of the TiC by self-propagating high-temperature synthesis. The microstructure of the resultant composites and the bonding interfaces was characterized by scanning electron microscopy and energy dispersive spectroscopy (SEM/EDS), X-ray diffraction (XRD), and transmission electron microscopy (TEM). The microstructural results showed a good adhesion of the composite, suggesting an effective infiltration of the metal into the inserted compact, yet a non-homogeneous distribution of the TiC in the martensite matrix was observed. Based on the results, the in situ synthesis appears to be a great potential technique for industrial applications.

## 1. Introduction

High-chromium white cast irons are based on the iron–chromium–carbon system. These alloys present an as-cast microstructure composed of a matrix of austenite (γ) or partially martensite (α’) with dispersed particles of iron–chromium carbides of M_7_C_3_ type. During the following destabilization heat treatment, secondary precipitation of fine M_23_C_6_ type carbides may occur together with the transformation of the γ into α’ [1,2,3,4].

The use of these alloys is widespread due to their high abrasive wear resistance. In particular, alloys with a Cr level ranging from 12% to 30% are applied for crushers, rollers, ball mill liners, and pulverizing equipment, the type of equipment used in the mineral, mining, and cement industries [2,5,6,7,8].

It should be noted that, even when applying hard materials, the abrasive wear mechanism is considered a critical and expensive problem in every processing industry, leading to the failure of components in service [9]. In this context, any single improvement in the wear resistance is a major achievement. Concerning the cast components, a possible approach to improve the wear resistance is to locally reinforce, with ceramic particles, the regions that will be exposed to wear while maintaining the toughness of the bulk component. In situ and ex situ methods could be applied to fabricate the composite reinforcement [10,11,12]. In the ex situ methods, the ceramic is previously produced with the required shape and then inserted into the mold [11,13,14,15,16,17,18,19,20,21,22], while in situ methods aim to produce the ceramic particles through the combustion reaction of the powder compacts inserted in the mold cavity [23,24,25,26,27,28].

During the casting process, the metal infiltrates into the pores of the powder compact. The heat of the molten metal causes the combustion reaction between the reactant powders, and the synthesis of the metal matrix composite reinforcement occurs [29,30].

The procedure of the in situ combustion synthesis (CS) is simple, low-cost, applicable to a wide range of geometries, and it permits producing particles with high purity [31]. Two ignition modes of CS are distinguished: self-propagating high-temperature synthesis (SHS) and thermal explosion (TE).

In the SHS mode, the combustion reaction is ignited by heating one end of the compact, beginning the exothermic reaction, which propagates through a combustion wave. With respect to the second case, the reaction is ignited by heating the whole compact, and the reaction occurs uniformly throughout the sample [27,32].

According to several investigations, titanium carbide (TiC) and titanium diboride (TiB_2_) are the best reinforcements for steel components due to their high hardness, excellent wear resistance, good wettability, and stability in ferrous matrices [12,33], although silicon, tungsten, and boron carbides are also reported to be effective reinforcements for wear applications [14,34,35].

Regarding the technology, investigations were focused on the CS of several powder systems, such as Ti–C [30,36,37,38,39,40], Ni–Ti–C [41,42], Ni–Ti–B_4_C [31], Fe–Ti–C/Fe–Cr–Ti–C [10,23,24,25,43,44], Cu–Ti–B_4_C [45,46,47], and Al–Ti–B_4_C [12,48]. Some of them were applied to the reinforcement of steel parts [12,24,25,42,43,45] and very few to the reinforcement of iron components [23,38,39,40].

The present research aims to investigate the local reinforcement of high-chromium white cast-iron specimens through TiC–high-chromium white iron matrix composites, fabricated in situ by SHS. This is of great practical value because high-chromium white cast irons are used for numerous applications that require high wear resistance. Powder mixtures of Ti and graphite were used with Al addition, because it is expected that Al will act as a deoxidizer and grain refiner, contributing to a decrease in the final porosity [49] and favoring the TiC particle size reduction [50].

The accurate characterization of the microstructural phases formed in the reinforced zone was the main objective of the present study since this specific topic was only slightly investigated until now.

## 2. Materials and Methods

To produce the reinforced specimens, a specific technique with several steps was applied. The first step was the selection and characterization of the initial powders. Commercial Ti powders (99.5 wt.% purity) from Alfa Aesar-ThermoFisher (Kandel) GmbH, Al powders (99.0 wt.% purity) from Goodfellow Cambridge Ltd., and graphite (99.0 wt.% purity) from Elsid (Snagov) S.A. were selected to prepare the green compacts. Scanning electron microscopy (SEM), using a FEI QUANTA 400 FEG (FEI Company, Hillsboro, OR, USA) with an energy-dispersive detector (EDS), and dynamic light scattering (DLS, Laser Coulter LS230 granulometer, Beckman Coulter, Inc., Brea, CA, USA) techniques were applied to analyze the morphology and granulometric distribution of the initial powders.

The Ti, Al, and graphite powders were mixed in a mass ratio of 64:20:16 and homogenized in a Turbula shaker-mixer (Willy A. Bachofen AG, Muttenz, Switzerland) for 7 h. Then, the whole mixture was cold-pressed at approximately 70 MPa in a metallic mold to produce parallelepipedal compacts of 31 mm × 12 mm × 7 mm. SEM analyses were performed to verify the quality of the powder mixture and green compacts.

At the end, the green compacts were inserted in the mold cavity, and the high-chromium white cast iron was poured at a temperature of 1460 °C. The chemical composition of the base metal is shown in Table 1.

**Figure 1:**
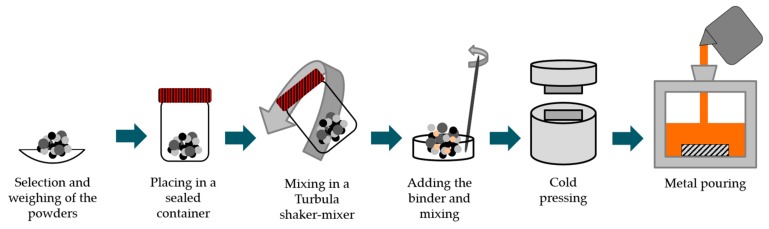
Scheme of the fabrication steps performed.

**Figure 2:**
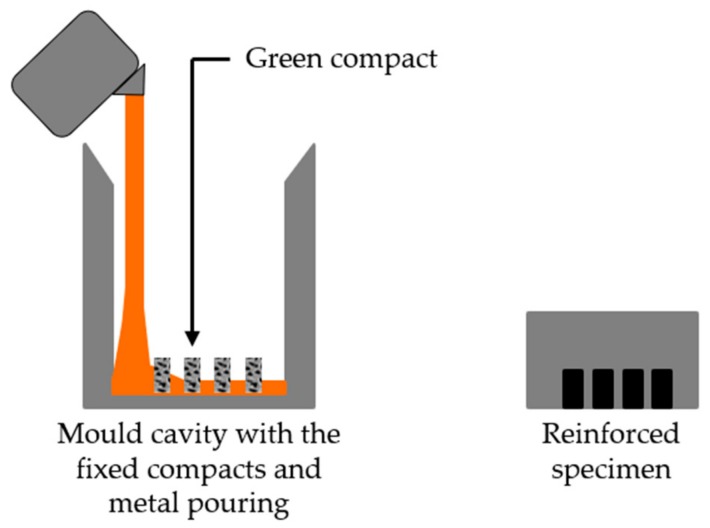
Scheme of the mold cavity with the inserted green compacts and the reinforced cast specimen.

The fabrication steps performed are shown in Figure 1. The scheme of Figure 2 presents the mold cavity with the inserted green powder compacts and a specimen locally reinforced.

The specimens were crosscut by wire electrical discharge machining to obtain metallographic samples that were ground and polished. After chemical etching with 2% Nital and Beraha-Martensite, the samples were characterized by optical microscopy (OM) using a Leica DM 4000M with a DFC 420 camera (Leica Microsystems, Wetzlar, Germany), SEM, and transmission electron microscopy (TEM) using a JEOL 2100 (JEOL Ltd., Akishima, Tokyo) operated at 200 keV. SEM images were obtained with secondary (SE) or backscattered (BSE) electron detectors. The size and content of the TiC particles in the reinforced specimens were measured from SEM-BSE images, using the image processing program ImageJ (version 1.52, Wayne Rasband, National Institutes of Health, Bethesda, MD, USA). The average of two diameters per particle was used to calculate the size of 500 particles, while the content of TiC was measured by the segmentation of 40 arbitrarily selected images.

A detailed characterization of the phases was performed using thin foils prepared in a dual-beam focused ion beam (FIB) FEI Helios NanoLab 450S (FEI Company, Hillsboro, OR, USA). On TEM, the phases were fully identified through selected area electron diffraction (SAED). Additionally, an energy dispersive X-ray spectrometer (EDS) coupled with the STEM mode was used to map the chemical composition of the phases, using the ZAF correction method.

X-ray diffraction (XRD, Cu Kα radiation, Bruker D8 Discover), with a scanning range (2ϴ) of 20° to 100°, was used to complement the characterization of the formed phases.

## 3. Results and Discussion

### 3.1. Characterization of the Starting Powders

**Figure 3:**
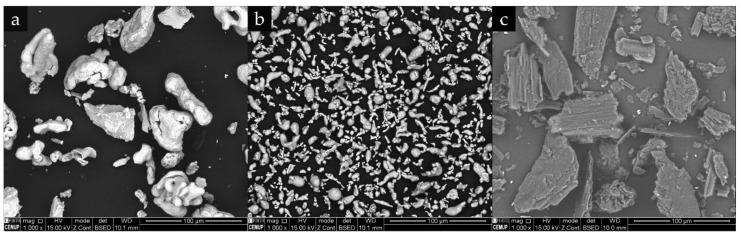
SEM-backscattered electron (BSE) images showing the morphology of the starting powders: (**a**) Ti, (**b**) Al, and (**c**) graphite.

**Figure 4:**
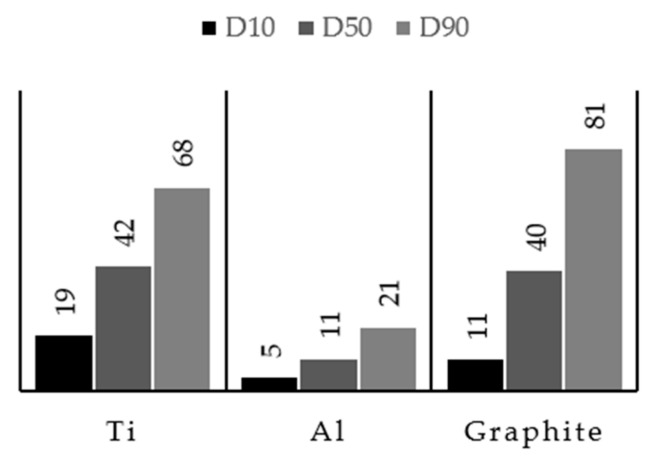
Particle size distribution (D_10_, D_50_, and D_90_) of starting powders. Data were collected from dynamic light scattering (DLS) analyses.

The morphology and the size distribution of the starting powders are presented in Figure 3 and Figure 4. From these figures, we can see that Ti, Al, and graphite powders exhibited different size, shape, and granulometric distribution. The Ti powders (see Figure 3a) presented an irregular shape and a larger size than Al powders. The results of the granulometric distribution showed an average size of 43 µm and a D_50_ of 42 µm, meaning that 50% of the particles were less than 42 µm (see Figure 4). It is interesting to point out that this result is in accordance with the supplier guide (average size of 44 µm). The Al powders also presented an irregular shape (Figure 3b); however, the majority of the particles were more elongated. In this case, the granulometric analysis (Figure 4) showed a D_50_ of 11 µm and an average size of 12 µm, being inferior to the average size of 25 µm, certified by the supplier. Lastly, the graphite powders, exhibiting a flake morphology (Figure 3c), were selected by a sieve separation and showed an average size of 43 µm and a D_50_ of 40 µm, in agreement with the adopted procedure.

**Figure 5:**
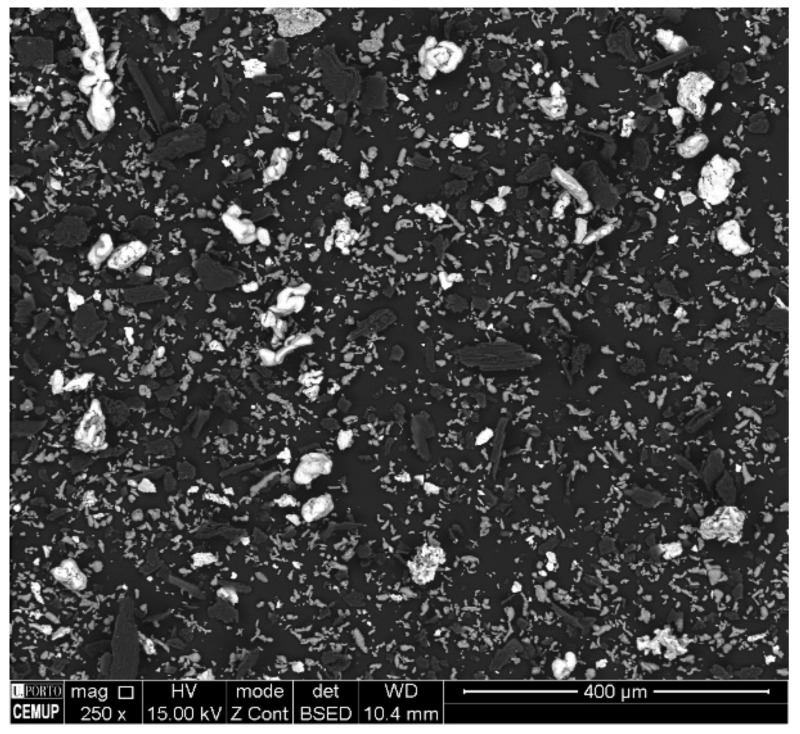
SEM-BSE image of the mixture of the starting powders and binder.

It is clear from Figure 5 that the mixing of the powders was well performed since the constituents were uniformly distributed without detecting the presence of agglomerates. There is evidence that the mixing step did not affect the morphology of the initial powders.

### 3.2. Characterization of the Green Powder Compacts

**Figure 6:**
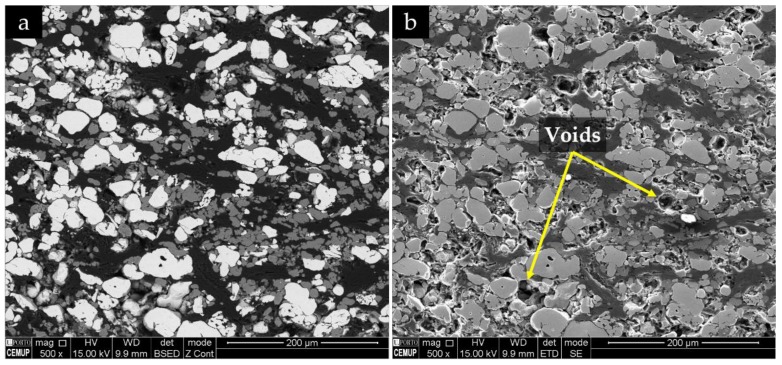
SEM images of the green powder compacts, using (**a**) BSE mode and (**b**) secondary electron (SE) mode.

SEM micrographs showing the morphology and the distribution of the initial powders in the green powder compacts are presented in Figure 6. The white particles correspond to Ti powders, the gray particles correspond to Al powders, and the dark regions correspond to the graphite or the binder phase (Figure 6a). Using an SE detector (Figure 6b), it was also possible to confirm the presence of voids in the structure. Actually, the existence of voids between the particles may be beneficial for the liquid metal infiltration.

### 3.3. Characterization of the Reinforced Specimens

**Figure 7:**
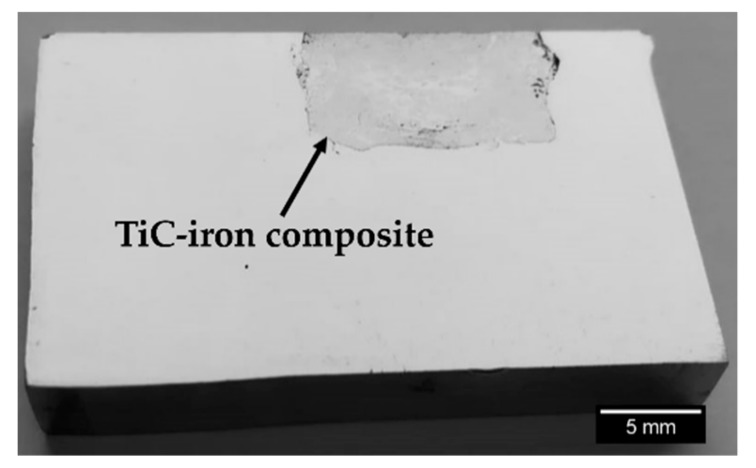
A polished cross-section of the reinforced specimen, showing the composite material.

A polished cross-section of the reinforced cast specimen is presented in Figure 7. It is possible to distinguish two zones, namely, the composite (gray zone) and the high-chromium white cast iron (light-gray zone). The composite zone presents a quasi-uniform depth (6 mm) and a width of around 12 mm. These dimensions are consistent with the initial dimensions of the green compact (12 mm × 7 mm).

#### 3.3.1. Base Metal

The chemical composition of the high-chromium white cast iron used in this research is presented in Table 1. According to the ASTM A532 standard [51], this alloy (25% Cr) is an abrasion-resistant cast iron of class III and type A.

**Figure 8:**
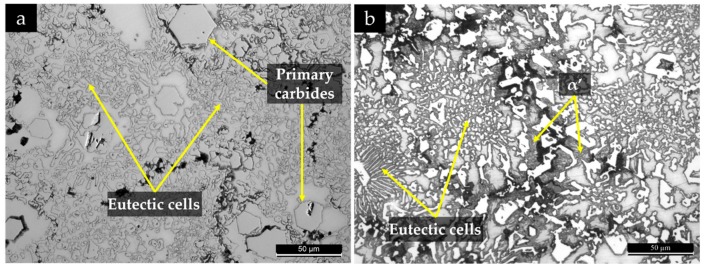
Optical image of the high-chromium white cast iron in the as-cast condition, after chemical etching with 2% Nital (**a**) and Beraha-Martensite (**b**).

Two different chemical etchings were used for the identification of the phases in the microstructure. The 2% Nital revealed large primary Cr-rich carbides with rod-like structure (white phase) and eutectic cells with finer Cr-rich carbides with blade-like structure surrounded by eutectic austenite (light-gray phase), as shown in Figure 8a. That is a typical microstructure of a hypereutectic ferrous alloy with a pro-eutectic phase (primary carbides) and eutectic cells formed from the eutectic reaction $L\;\to \;\gamma +{\left(Fe,\;Cr\right)}_{7}\;C_{3}$ [1,52]. The acicular α’ was revealed by etching with Beraha-Martensite, as shown in Figure 8b.

**Figure 9:**
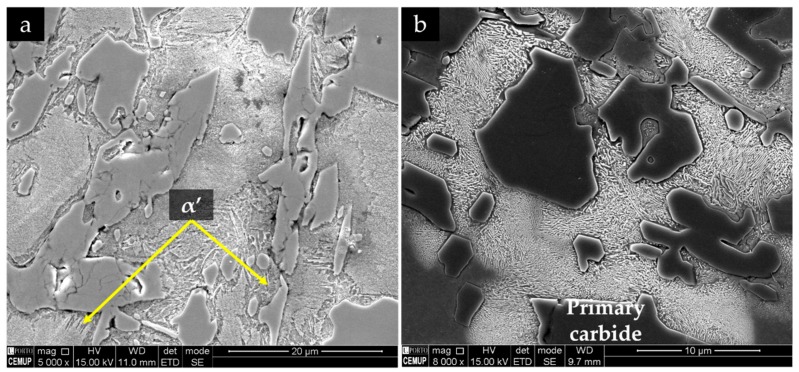
SEM-SE image of the microstructure of the high-chromium white cast iron in the as-cast condition at different magnifications.

The achievement of a fully austenitic matrix depends on the chemical homogeneity and the cooling rate imposed; for instance, a localized Cr and C depletion due to carbide formation may provoke the transformation of γ to α’ because of the increase in martensite start temperature (Ms) [5,6]. The dark phase observed in Figure 8 could not be clarified in optical microscopy; thus, SEM and XRD characterization were undertaken to evaluate the microstructure in detail. High-magnification SEM observations showed a lamellar constituent, possibly bainite (see Figure 9).

**Figure 10:**
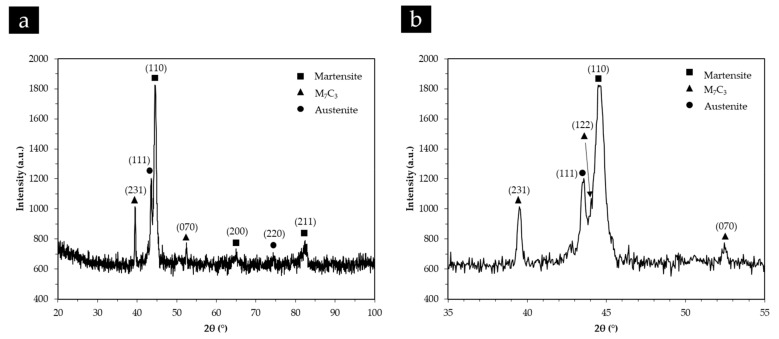
X-ray diffraction (XRD) patterns of the high-chromium white cast iron in the as-cast condition in the 2ϴ range of 20°–100° (**a**) and 35°–55° (**b**).

The XRD analyses confirmed the presence of Cr-rich carbides (M_7_C_3_), γ and α’, as shown in the XRD patterns present in Figure 10, which is consistent with other reported results for white cast irons with more than 10% Cr [5,52]. However, no other phase was detected in these patterns that could be associated with the lamellar constituent identified from the SEM images, possibly explained by the low content of this phase.

#### 3.3.2. Composite Zone

**Figure 11:**
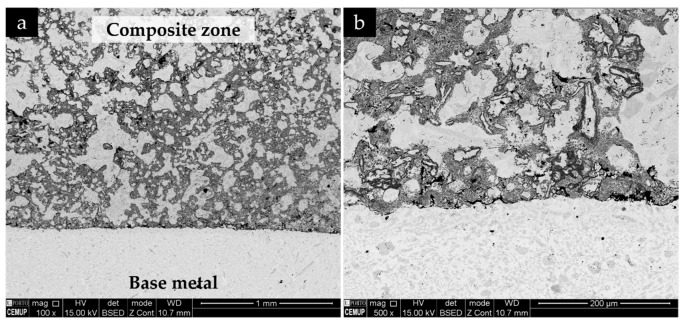
SEM-BSE images of the microstructure of the reinforced specimen: (**a**) base metal and composite and (**b**) interface region at higher magnification.

**Figure 12:**
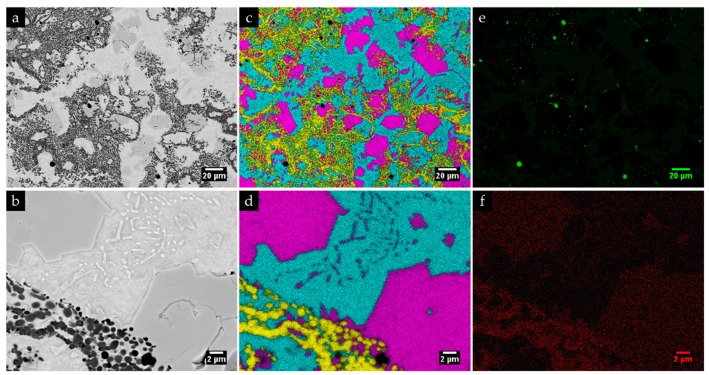
SEM-BSE images of the microstructure of the composite zone (**a**,**b**) at higher magnification; energy-dispersive spectroscopy (EDS) elemental mapping: (**c**,**d**) superposition of Cr (pink), Ti (yellow), and Fe (blue); (**e**) mapping of Al and (**f**) mapping of C.

The microstructure of the composite and the interface is presented in Figure 11. SEM images show a sound bonding between the composite and the metal matrix with no evidence of voids and porosities, suggesting a good infiltration of the metal into the inserted green powder compact. One possible explanation is the melting of the Al that improves the bonding between the composite and the base metal [50]. The EDS/EDX maps presented in Figure 12 show Ti rich-zones (in yellow) corresponding to titanium carbide networks, Cr-rich zones (in pink) associated to the pro-eutectic and eutectic Cr carbides, and Fe-rich zones (in blue) matching the metal matrix. Figure 12e points to the presence of Al rich-zones that corresponded to the aluminum oxide particles, in green. These oxides could come from the reaction of the Al with the oxygen, during the casting process. At higher magnification (see Figure 12d), fine carbide precipitates, presumably eutectic carbides, were identified in the matrix.

**Figure 13:**
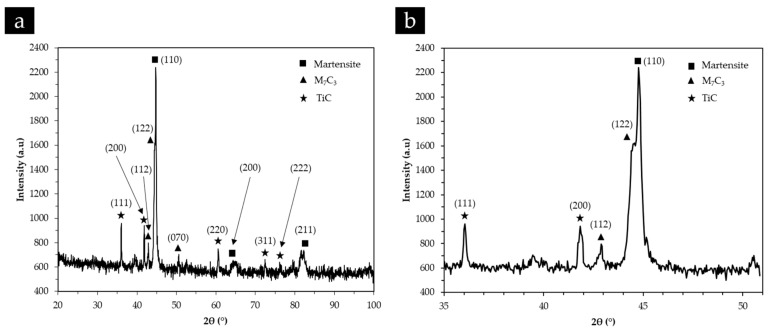
XRD patterns of the composite zone in the 2ϴ range of 20°–100° (**a**) and 35°–51° (**b**).

Figure 11 and Figure 12 also show a non-homogeneous distribution of titanium carbide networks in the white cast iron matrix. Some authors [37,39] described this structure as a cellular structure similar to that observed in metallic foams. The XRD analysis (see Figure 13) permitted identifying the presence of TiC, which confirms the effectiveness of the in situ TiC synthesis, martensite, and M_7_C_3_ Cr-rich carbides. To note, titanium aluminides (TiAl_x_ (x = 1, 3)) were not identified in this analysis, possibly indicating that the combustion reaction of the Ti and graphite powders was complete, similarly to that referred by Song et al. [27]. It should be pointed out that the diffractograms do not show any peak of γ, in contrast to those relative to the base metal (Figure 10).

**Figure 14:**
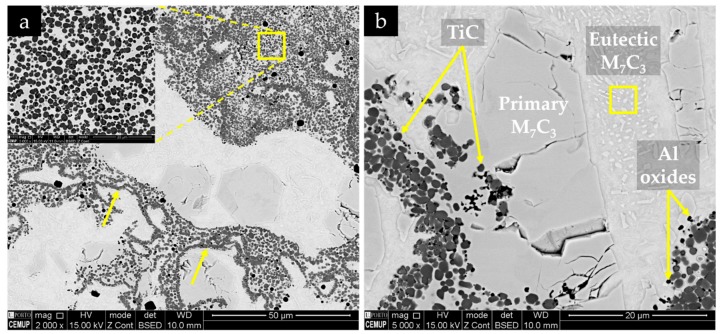
SEM-BSE images of the microstructure of the composite zone, showing (**a**) TiC particle clusters, highlighted in the image; (**b**) with higher magnification, coarse primary and eutectic M_7_C_3_ carbides, and Al oxides (black particles).

Higher-magnification SEM analysis was used to characterize the morphology of the formed phases. Figure 14 shows the TiC particles embedded in the metallic matrix. These particles assume a nearly spherical morphology, and some agglomerates are noted. Figure 14 also exhibits lines of TiC particles (indicated by the yellow arrows). According to Olejnik et al. [38], the formation of these agglomerates results from the bonding of single TiC particles into large aggregates, which take the form of “bubbles” that are filled with the liquid base metal.

**Figure 15:**
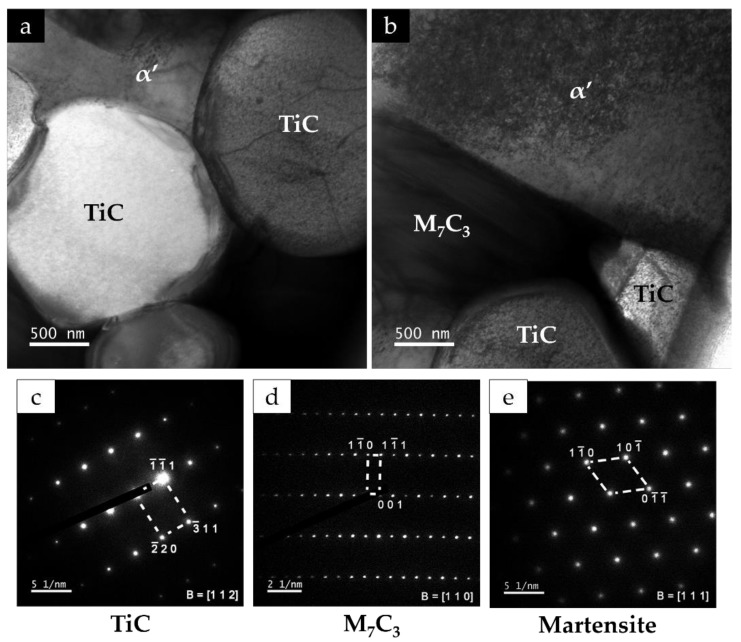
Dark-field TEM image of the composite showing (**a**) TiC particles and α’ phase and (**b**) interface between α’ and a M_7_C_3_ type carbide. The identification of phases was conducted by selected area electron diffraction (SAED) analysis with (**c**) [1 1 2] zone axis of TiC, (**d**) [1 1 0] zone axis of M_7_C_3_, and (**e**) [1 1 1] zone axis of α’.

**Figure 16:**
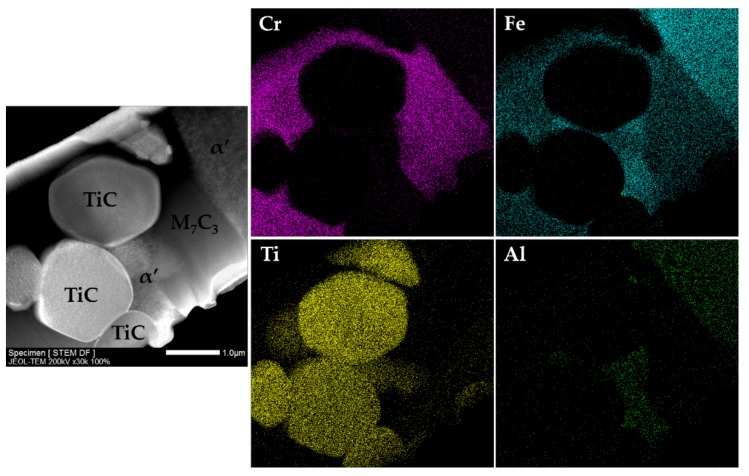
Dark-field STEM image of the composite zone and the EDS maps of Cr, Fe, Ti, and Al from the correspondent area.

In addition, the TEM/SAED and dark-field STEM analysis of the composite zone confirmed the in situ formation of round particles of TiC and a matrix of α’ with Cr-rich carbides (M_7_C_3_) precipitated (see Figure 15 and Figure 16). Figure 15a evidences two round TiC particles with different orientation embedded in a matrix of α’ with large M_7_C_3_ carbides, and Figure 15b exhibits an interface between α’ and a large M_7_C_3_ carbide. It was also possible to notice agglomerated particles, as shown in Figure 16.

**Figure 17:**
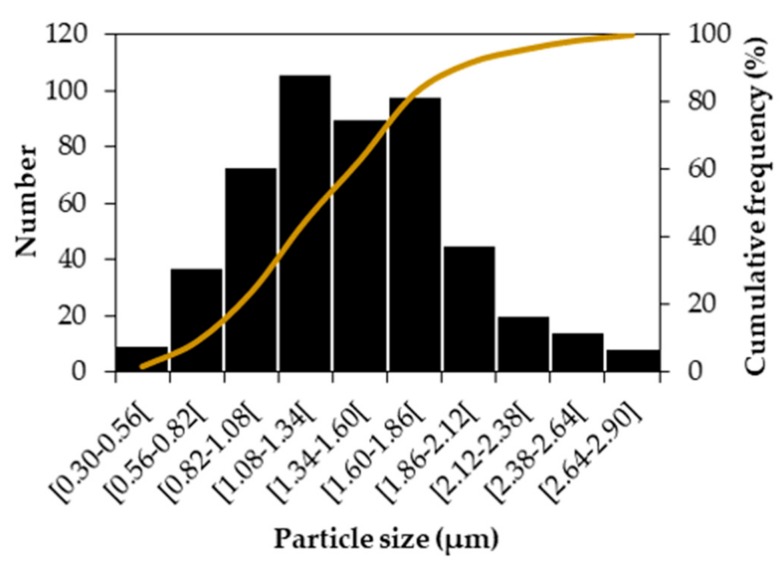
Size distribution of the TiC particles in the composite zone (two perpendicular diameters were measured in 500 particles).

The characterization of the composite zone was concluded by measuring the size and content of TiC particles. The TiC content, measured by image segmentation of SEM-BSE images taken on 40 randomly selected fields at a magnification of 2000×, showed a variation from 1% to 43% and an average of 24%, which confirmed the low homogeneity of the TiC particles distribution. The quantitative data obtained concerning the size of the TiC particles are depicted in Figure 17. The histogram shows a normal distribution, and a minimum and maximum value of 0.30 µm and 2.90 µm. The cumulative frequency curve shows that 50% of the analyzed particles were smaller than 1.34 µm. These results are in line with those reported by He et al. [23] on a TiC–Fe-based composite produced by SHS.

## 4. Conclusions

High-chromium white cast-iron specimens locally reinforced with TiC–metal matrix composites were successfully produced via an in situ technique based on combustion synthesis, using powder mixtures of Ti, Al, and graphite.

The TiC–white cast-iron composite was composed of TiC particles embedded in a matrix of α’ with rod- and blade-shaped Cr-rich carbides (M_7_C_3_). The absence of voids and porosities suggests a good infiltration of the liquid metal into the inserted green powder compact.

The average content of TiC particles in the composite zone was 24%. The majority of the TiC particles assumed a nearly spherical morphology and a size inferior to 1.34 µm.

The findings of this study suggest, therefore, that the in situ synthesis appears to be a great potential technique for high-chromium white cast-iron applications.

## Figures and Tables

**Table 1:** Chemical composition of the studied high-chromium white cast iron (wt.%).

C	Si	Mn	Cr	Ni	Fe
3.10	0.60	0.54	26.80	0.30	Balance
